# Eight-Year Clinical Outcomes of Transcatheter Aortic Valve Replacement with J-Valve System

**DOI:** 10.5761/atcs.oa.24-00152

**Published:** 2025-05-03

**Authors:** Fei Li, Yuetang Wang, Donghui Xu, Xu Wang, Wei Wang

**Affiliations:** 1 Heart Valve and Atrial Fibrillation Center, Beijing Anzhen Hospital, Capital Medical University, Beijing, China; 2 Department of Structural Heart Disease, Fuwai Hospital, Chinese Academy of Medical Sciences, Beijing, China

**Keywords:** transcatheter aortic valve replacement, structural valve deterioration, aortic stenosis, pure aortic regurgitation, long-term follow-up

## Abstract

**Purpose:** This study aimed to summarize 8-year clinical outcomes for patients who underwent transcatheter aortic valve replacement (TAVR) with the J-Valve system and evaluate the long-term durability and hemodynamic performance of the valve.

**Methods:** Between July 2014 and June 2015, 21 patients underwent transapical TAVR with the J-Valve system. Systematic clinical and echocardiographic follow-up was conducted on 18 patients for up to 8 years.

**Results:** Eight years post-TAVR with the J-Valve system, the all-cause mortality rate was 16.7%, with no prosthesis failures or thrombosis. Moderate to severe valve deterioration was observed in 50% of patients with aortic stenosis (AS), whereas no such deterioration was noted in patients with pure aortic regurgitation (PAR). At 8 years following TAVR, the effective orifice area measured 2.27 ± 0.50 cm^2^ in patients with PAR and 1.35 ± 0.38 cm^2^ in those with AS. Additionally, patients with AS exhibited a mean pressure gradient of 17.90 ± 10.61 mmHg. Over 8 years, PAR patients experienced a significant reduction in left ventricular end-diastolic diameter from 61.50 ± 2.08 mm to 48.67 ± 7.23 mm (*p* < 0.001), whereas AS patients showed no significant change.

**Conclusion:** The J-Valve system demonstrates favorable long-term outcomes in TAVR, with excellent durability and hemodynamic performance in PAR patients.

## Introduction

Transcatheter aortic valve replacement (TAVR) is frequently employed as an alternative to surgical aortic valve replacement in the management of severe aortic stenosis (AS) with low surgical risk patients, and for the treatment of severe pure aortic regurgitation (PAR) in patients deemed medically inoperable.^1,2)^ TAVR now achieves clinical outcomes at 5 years that are comparable to those of surgical intervention in patients with severe aortic stenosis.^2,3)^ The J-Valve system, an advantageous “on-label” transcatheter heart valve (THV), has been shown to effectively treat both PAR and AS.^4–6)^ In previous studies,^5)^ we confirmed its superior clinical performance and valve durability over a 4-year follow-up period.

Over the past decade, the adoption of TAVR has been principally propelled by a robust corpus of clinical evidence derived from randomized controlled trials.^1,7)^ This evidence has facilitated the progressive extension of TAVR to encompass lower-risk and younger patient populations.^2)^ TAVR has progressed beyond its original role as a mere remedial intervention,^1,7)^ necessitating prolonged monitoring for periods exceeding 5 years. This extended surveillance is particularly crucial as TAVR is increasingly employed in lower-risk, younger patient populations who are projected to have longer life expectancies. To date, research has extensively documented the long-term clinical outcomes, extending over 8 years and beyond, in patients with AS who have undergone TAVR with various types of THVs.^8,9)^ However, there is a notable absence of reports concerning very long-term follow-up on clinical and valve performance outcomes for patients with PAR. Building upon the previously collected 4-year follow-up data from the clinical trial conducted at our center,^5)^ this study aims to present the 8-year clinical outcomes of patients who underwent TAVR using the J-Valve.

## Materials and Methods

### Participants and TAVR procedures

Patients diagnosed with severe PAR or AS and identified as high surgical risk were consecutively enrolled at Fuwai Hospital from July 2014 to June 2015. The criteria for patient selection were comprehensively delineated in our previous study.^4)^ Among the 21 patients who underwent transapical TAVR with the J-Valve, 18 patients (14 with AS and 4 with PAR) were followed up. Clinical and echocardiographic data were collected over a follow-up period extending up to 9 years.

The J-Valve THV consists of a porcine aortic valve affixed to a cylindrical, self-expanding, nitinol stent, which is further equipped with three U-shaped graspers integrated into the stent structure. Prior to deployment, these graspers are released and anatomically aligned with the Valsalva sinuses to serve as landmarks. The implantation procedure has been comprehensively described in our prior publications.^4)^

### Definitions of structural valve deterioration

The aortic valve was assessed through two-dimensional echocardiography to evaluate its morphological structure, quantify paravalvular leakage, measure the trans-aortic mean gradient, and calculate the effective orifice area. Multi-detector computed tomography was employed to investigate suspected valve thrombosis identified during the echocardiographic examination. Paravalvular leakage and intra-prosthetic aortic regurgitation were classified into categories of none, mild, moderate, or severe. Hemodynamic and morphological structural valve deterioration (SVD) were defined according to the standardized criteria established by EAPCI/ESC/EACTS.^10)^

### Follow-up schedule

The follow-up protocol for the clinical trials of the J-Valve was established prior to its market release, mandating an initial follow-up period of 5 years for all patients. Upon completion of this 5-year period,^4)^ the follow-up duration was extended to 8 years, with approval from the local ethics committee. Informed consent was obtained from all patients prior to their enrollment in the study. Follow-up data on clinical status, adverse events, survival, New York Heart Association (NYHA) classification, and echocardiographic data were systematically collected at the time of discharge, followed by subsequent assessments at 1 month, 6 months, and annually up to 5 years, as well as at 8 years, through scheduled outpatient visits and telephone interviews.

### Statistical analysis

Continuous variables were assessed for normality using the Shapiro–Wilk test. Based on their distribution, these variables were either reported as mean ± standard deviation or median (interquartile range). Categorical variables were expressed as frequency (*n*) and percentage (%). To evaluate the differences in mean aortic valve gradient and aortic valve orifice area across multiple time points, either one-way ANOVA or the Kruskal–Wallis rank sum test was applied, depending on the data distribution. Post hoc comparisons were executed utilizing the Bonferroni correction method. All statistical analyses were carried out using SPSS software, version 29.0.1.0 (IBM Corp., Armonk, NY, USA).

## Results

### Baseline and procedural characteristics

The baseline and procedural characteristics are comprehensively delineated in **Table 1**. Most patients (13, 72.2%) were male. The mean age was 74.07 ± 4.87 years for patients with AS and 76.00 ± 6.88 years for patients with PAR. Among the AS patients, 50.0% had a tricuspid aortic valve, whereas all patients with PAR had a tricuspid aortic valve. Upon admission, 13 patients in the AS cohort and 3 patients in the PAR cohort were classified as having NYHA functional class III/IV. The average society of thoracic surgeons mortality score for AS patients was 9.43 ± 2.64, while the score for PAR patients was 9.94 ± 1.17.

**Table 1:** Baseline characteristics

Characteristics	AS (*n* = 14)	PAR (*n* = 4)
Demographics		
Male sex, *n* (%)	10 (71.4)	3 (75.0)
Age (year)	74.07 ± 4.87	76.00 ± 6.88
BMI	24.90 ± 3.98	25.07 ± 3.05
Aortic valve phenotypes, *n* (%)		
BAV	7 (50.0)	0 (0)
TAV	7 (50.0)	4 (100.0)
Medical history, *n* (%)		
Prior heart surgery	1 (7.1)	0 (0)
Prior stroke	6 (42.9)	1 (25.0)
COPD	2 (14.3)	1 (25.0)
Laboratory tests		
Creatinine (μmol/L)	82.04 (66.22–95.23)	70.69 ± 18.99
eGFR (mL/minute)	58.65 ± 22.45	65.31 ± 5.85
ALT (U/L)	18.64 ± 3.61	18.75 ± 0.96
AST (U/L)	17.29 ± 5.05	18.25 ± 7.50
ALP (U/L)	58.57 ± 17.86	65.75 ± 13.62
Albumin (g/L)	39.16 ± 2.90	39.68 ± 2.31
NT-proBNP (pg/mL)	932.65 (405.33–2204.50)	412.15 (192.78–2093.95)
Cardiovascular comorbidity, *n* (%)		
Coronary artery disease	8 (57.1)	1 (25.0)
Atrial fibrillation	1 (7.1)	1 (25.0)
Risk scores		
STS score	9.43 ± 2.64	9.94 ± 1.17
Functional status, *n* (%)		
NYHA functional class III/IV	13 (92.9)	3 (75.0)

Based on data normality, continuous variables were reported as mean ± standard deviation or median (interquartile range); categorical variables were presented as number (n) and percentage (%). BMI: Body Mass Index; BAV: bicuspid aortic valve; COPD: chronic obstructive pulmonary disease; TAV: tricuspid aortic valve; STS: Society of Thoracic Surgeons; NYHA: New York Heart Association

The procedural characteristics are comprehensively detailed in our previous study.^5)^ In summary, the mean duration of hospitalization was 21.6 days, the average length of stay in the intensive care unit was 50.6 hours, and the mean duration of ventilator support was 30.7 hours. The J-Valve prosthesis was successfully implanted during the initial procedure for 13 patients with AS and 4 patients with PAR. One patient with AS experienced embolization of the THV to the ascending aorta, which required the implantation of a second prosthesis in the correct position. In patients with AS, none or trivial paravalvular leakage was observed in six patients (42.9%), while mild leakage was noted in eight patients (57.1%) during the immediate postoperative period. Notably, no instances of moderate or severe leakage were detected. All patients with PAR exhibited none or trivial paravalvular leakage.

### Clinical follow-up outcomes

**Fig. 1:**
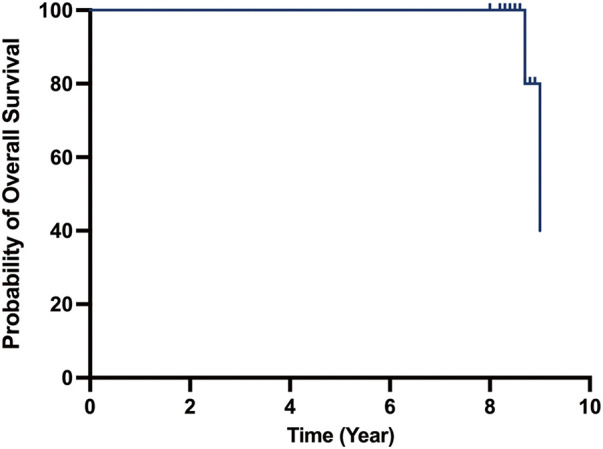
Kaplan–Meier survival curves for all 18 patients

Patients were monitored for a duration of up to 9 years postoperatively through regularly scheduled clinic visits. During the follow-up period, two patients with AS and one patient with PAR succumbed. **Figure 1** shows the Kaplan–Meier survival curves. Specifically, one patient with AS died from chronic renal failure 8 years following TAVR, while another patient with AS succumbed to gastrointestinal bleeding 8.5 years post-procedure. Additionally, a patient diagnosed with PAR passed away due to myocardial infarction at 8.9 years post-implantation. Reinterventions following TAVR were observed in two cases of AS due to progressive stenosis, occurring at 5.3 and 6.5 years post-procedure, respectively. Notably, no patients with PAR necessitated reintervention after TAVR. One patient with AS developed a late-onset complete atrioventricular block.

**Fig. 2:**
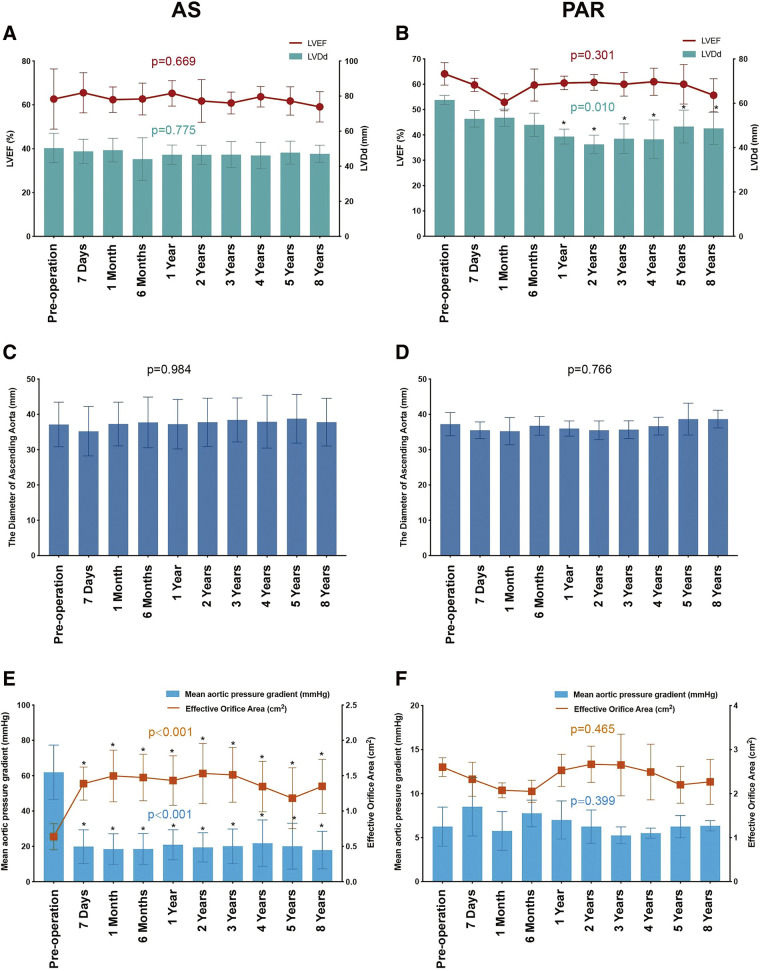
Echocardiographic characteristics were evaluated preoperatively, immediately post-implantation, and throughout the follow-up period. **(A, B)** Left ventricular injection fraction and left ventricular end diastolic dimension. **(C, D)** The diameter of ascending aorta. **(E, F)** Mean trans-aortic pressure gradient and aortic valve orifice area. *Indicates a statistically significant difference (*p* <0.05) when compared with preoperative measurements. LVDd: left ventricular diastolic dimension

Among the surviving patients, 12 were classified as NYHA functional class I/II at the 8-year follow-up. No significant difference in left ventricular ejection fraction was observed between the preoperative period and each follow-up visit in both AS (*p* = 0.669) and PAR patients (*p* = 0.301; **Figs. 2A**, **2B**). The left ventricular diastolic dimension (LVDd) demonstrated a statistically significant reduction in patients with PAR 1 year following TAVR (*p* = 0.010; **Fig. 2B**). Conversely, no significant alteration in LVDd was detected in patients with AS (*p* = 0.775; **Fig. 2A**). Furthermore, the diameter of the ascending aorta remained constant regardless of the type of valve dysfunction (AS: *p* = 0.984; PAR: *p* = 0.766; **Figs. 2C**, **2D**).

### Hemodynamic performance and valve durability

**Fig. 3:**
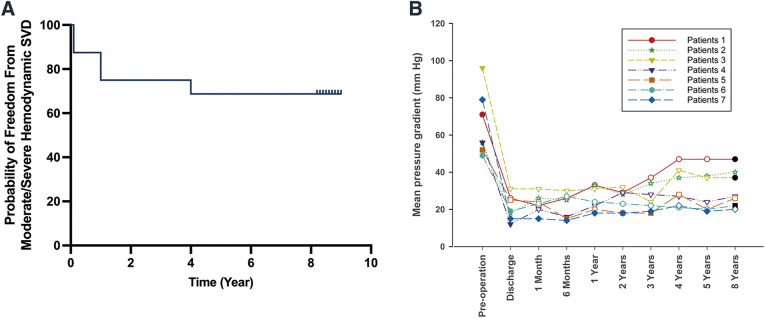
(**A**) Kaplan–Meier curves for freedom from moderate/severe hemodynamic SVD in AS patients. **(B)** The mean aortic pressure gradient from preoperative assessment through to the 8-year follow-up for seven patients diagnosed with SVD was illustrated. Symbols filled with white represented the occurrence of moderate SVD at specific time points, and those filled with black indicated severe SVD occurrences. SVD: structural valve deterioration, AS; aortic stenosis

**Fig. 4:**
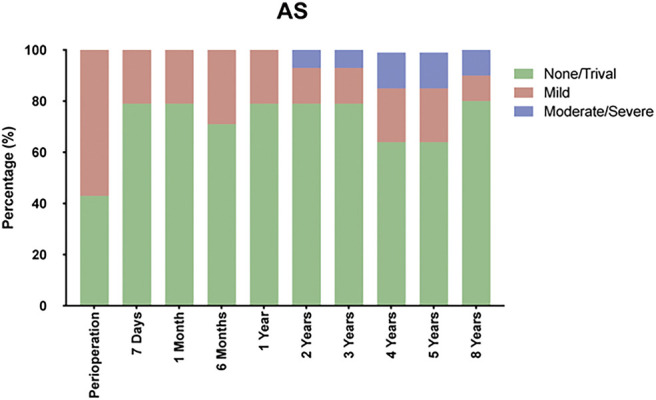
Paravalvular regurgitation at all evaluated time points

Echocardiographic evaluations were conducted at each visit during the follow-up period. No instances of prosthetic valve thrombosis or morphological SVD were detected. Among patients with AS, the mean aortic pressure gradients significantly decreased following the TAVR procedure (*p* < 0.001), with no significant changes observed across subsequent follow-up assessments (**Fig. 2E**). No statistically significant difference was observed in the mean aortic pressure gradients between the preoperative and postoperative measurements in patients with PAR (*p* = 0.399; **Fig. 2F**). The mean effective orifice area for patients with AS was 1.35 ± 0.38 cm^2^ at 8 years post-TAVR, with no significant decrease observed across the follow-up period (**Fig. 2E**). In patients with PAR, the mean effective orifice area did not demonstrate statistical significance across the various time points measured (*p* = 0.465; **Fig. 2F**). According to the standardized definitions of SVD and THV failure by EAPCI/ESC/EACTS,^10)^ seven patients with AS exhibited moderate to severe hemodynamic SVD at the 8-year follow-up. Of them, two patients with severe hemodynamic SVD underwent re-intervention. **Figure 3A** illustrates Kaplan–Meier curves for freedom from moderate/severe hemodynamic SVD in patients with AS. No occurrences of moderate to severe hemodynamic SVD were identified in patients with PAR. The mean aortic pressure gradient for each patient (*n* = 7) diagnosed with moderate/severe hemodynamic SVD is depicted in **Fig. 3B**. Among patients with AS, 80.0% demonstrated none/trival paravalvular regurgitation at the 8-year follow-up (**Fig. 4**). Throughout the entire follow-up period, no instances of mild or greater paravalvular regurgitation were observed in patients with PAR.

## Discussion

These findings substantiate the very long-term efficacy of the J-Valve in patients with both PAR and AS over an 8-year follow-up period. Notably, no morphological or hemodynamic SVD of the J-Valve was observed in patients with PAR. Furthermore, LVDd significantly decreased 1-year post-TAVR, underscoring the valve’s excellent therapeutic efficacy for PAR. At the 8-year follow-up, seven patients with AS demonstrated moderate to severe hemodynamic SVD. Among these patients, two underwent repeat TAVR, while the remaining five exhibited no significant clinical symptoms. Late-onset complete atrioventricular block was observed in only one patient with AS. Importantly, no morphological SVD was reported in the AS cohort, underscoring the long-term safety and efficacy of the J-Valve in this population.

PAR poses several anatomical challenges to the implementation of TAVR.^11)^ One significant issue is the lack of substantial aortic valve calcification,^12)^ which is crucial for secure anchoring of the THV. Moreover, severe PAR is frequently associated with the enlargement of the aortic valve annulus and dilation of the aortic root,^13)^ further complicating the procedure. Additionally, the “suction effect” induced by regurgitation can destabilize TAVR deployment, thereby increasing the risk of THV migration or embolization. Many THVs, such as the Medtronic CoreValve^14)^ and Evolut R,^15)^ Edwards Sapien XT and 3,^16)^ and Acurate Neo,^17)^ were initially designed for the treatment of AS but are also utilized for off-label indications in the management of PAR.^18,19)^ Research indicates that first-generation “off-label” THVs utilized in TAVR procedures demonstrate suboptimal procedural success rates in patients with PAR.^18,19)^ Second-generation “off-label” THVs have exhibited enhanced efficacy in TAVR for PAR relative to their first-generation predecessors.^19)^ However, the procedural success rates of these second-generation “off-label” THVs remain inferior to those achieved with “on-label” THVs specifically approved for use in PAR, such as the J valve and JenaValve.^18)^

The design of these two “on-label” devices has progressed to a new generation, transitioning the access method from transapical to transfemoral.^20–23)^. Compared with early-generation “on-label” THVs, the new generation of Jena Valve and J-Valve demonstrated technical success rates of 95%^20)^ and 81%,^22)^ respectively. Furthermore, the latest design iterations of the Jena Valve and J-Valve exhibited promising short-term clinical and hemodynamic outcomes, with a 1-year post-TAVR mortality rate of 7.8% for the Jena Valve^20)^ (*n* = 180) and only one death at 30 days for the J-Valve^22)^ (*n* = 27). These specialized THVs present significant advantages in procedural efficacy and safety for the treatment of moderate-to-severe PAR.

The distinctive design of the “on-label” THVs includes a valve-locating feature comprising three radiopaque locators, specifically engineered to conform to the native aortic valve sinuses.^21,23)^ This design seeks to restrict the implant depth and attach to the native leaflets, thereby offering an anchoring mechanism and improving the seal surrounding the THV. Nonetheless, certain subsets of PAR remain therapeutic challenges, including conditions such as bicuspid aortic valve, an enlarged aortic annulus, dilation of the aortic root, and horizontal aortas. Type-0 bicuspid aortic valve, characterized by the presence of two aortic sinuses and two leaflets without a raphe,^24)^ does not conform to the three symmetrically distributed locators on “on-label” THVs. This misalignment frequently leads to localization failure or results in the excessively elevated positioning of the THV. Additionally, dilatation or enlargement of the aortic sinus, commonly observed in PAR patients, may lead to incomplete attachment of the clip to an individual leaflet due to lateral displacement of the locator.

The incidence of moderate/severe hemodynamic SVD observed in our study was significantly higher compared with the findings reported in other studies. Specifically, in our cohort of patients with AS, moderate/severe hemodynamic SVD was identified in 21.4% (3/14) of cases at discharge. This rate increased to 42.8% (6/14) at the 4-year follow-up and further increased to 50.0% (7/14) at the 8-year follow-up. In contrast, Testa et al.^25)^ reported an incidence of moderate/severe SVD of 3.0% among surviving patients at 8 years, while Didier et al.^26)^ documented an incidence of 13.3% in their cohort of surviving patients. It is important to highlight that the 8-year all-cause mortality rate was 78.3% and the 5-year all-cause mortality rate was 60.8% in the study conducted by Testa and Didier. These rates are significantly higher than the all-cause mortality rate observed in AS patients within our study, which was 14.3% (2/14). It is unequivocal that patients experiencing moderate to severe hemodynamic SVD following TAVR are at an elevated risk of mortality compared with those without such conditions. Mortality may serve as a competing risk against valve failure over time, potentially resulting in significant discrepancies in the reported assessments of SVD.

This study presents the first 8-year follow-up data on the J-Valve, highlighting its substantial clinical efficacy, especially for patients with PAR. TAVR with the J-Valve is recommended as the preferred treatment over surgical aortic valve replacement for high-risk patients with PAR.

### Limitation

The sample size in this study was limited; thus, additional cases are necessary to validate our findings.

## Conclusion

The extended durability and exceptional hemodynamic performance of the J-Valve offer substantial evidence supporting the widespread implementation of TAVR in patients with PAR. In patients with AS, the J-Valve showed consistent safety and therapeutic effectiveness over an 8-year follow-up.

## Declarations

### Ethics approval and consent to participate

This study was approved by the local ethical committee of Fuwai Hospital (Approval No.: 2024-2285), and all patients signed informed consent before enrollment.

### Consent for publication

All authors have given their consent for publication.

### Funding

None.

### Disclosure statement

The authors declare that they have no conflicts of interest.

### Data availability

Data are available on request from the corresponding author.

### Authors’ contributions

Fei Li and Yuetang Wang made substantial contributions to patient follow-up, manuscript drafting, and the execution of statistical analyses.

Xu Wang, Wei Wang, Donghui Xu undertook the TAVR procedures. All authors edited, reviewed, and approved the final manuscript.

All authors have made substantial contributions to the work, warranting public responsibility for its content.

Furthermore, they have consented to be accountable for the accuracy and integrity of the manuscript.
